# RclS Sensor Kinase Modulates Virulence of *Pseudomonas capeferrum*

**DOI:** 10.3390/ijms23158232

**Published:** 2022-07-26

**Authors:** Katarina Novović, Milka Malešević, Miroslav Dinić, Lazar Gardijan, Milan Kojić, Branko Jovčić

**Affiliations:** 1 Institute of Molecular Genetics and Genetic Engineering, University of Belgrade, Vojvode Stepe 444a, 11042 Belgrade, Serbia; milkam@imgge.bg.ac.rs (M.M.); mdinic@imgge.bg.ac.rs (M.D.); gardijan@imgge.bg.ac.rs (L.G.); mkojic@imgge.bg.ac.rs (M.K.); Serbia; bjovcic@bio.bg.ac.rs (B.J.); 2 Faculty of Biology, University of Belgrade, Studentski Trg 16, 11000 Belgrade, Serbia

**Keywords:** three-component system, sensor kinase, *Pseudomonas*, RNA sequencing, antibiotic resistance, virulence

## Abstract

Signal transduction systems are the key players of bacterial adaptation and survival. The orthodox two-component signal transduction systems perceive diverse environmental stimuli and their regulatory response leads to cellular changes. Although rarely described, the unorthodox three-component systems are also implemented in the regulation of major bacterial behavior such as the virulence of clinically relevant pathogen *P. aeruginosa*. Previously, we described a novel three-component system in *P. capeferrum* WCS358 (RclSAR) where the sensor kinase RclS stimulates the *intI1* transcription in stationary growth phase. In this study, using *rclS* knock-out mutant, we identified RclSAR regulon in *P. capeferrum* WCS358. The RNA sequencing revealed that activity of RclSAR signal transduction system is growth phase dependent with more pronounced regulatory potential in early stages of growth. Transcriptional analysis emphasized the role of RclSAR in global regulation and indicated the involvement of this system in regulation of diverse cellular activities such as RNA binding and metabolic and biocontrol processes. Importantly, phenotypic comparison of WCS358 wild type and Δ*rclS* mutant showed that RclS sensor kinase contributes to modulation of antibiotic resistance, production of AHLs and siderophore as well as host cell adherence and cytotoxicity. Finally, we proposed the improved model of interplay between RclSAR, RpoS and LasIR regulatory systems in *P. capeferrum* WCS358.

## 1. Introduction

Signal transduction systems are the eyes and ears of bacterial cells that perceive diverse extracellular stimuli and enable the adequate cellular response [1]. Bacterial cells are in continuous struggle with dynamic changes in the environment; thus both sensing of fluctuating signals and adequate and fast response are crucial for surviving and thriving of the bacterial cell [1]. The most common form of bacterial signal transduction systems is the two-component system (TCS) consisting of the membrane-associated sensor histidine kinase and its cognate cytoplasmic response regulator, which is usually the activator of gene transcription [1]. In addition, some response regulators are the effectors that interact with RNAs or proteins or even have an enzymatic activity [2]. Response regulators with enzymatic effector domains are commonly involved in the intracellular level regulation of key cellular alarmons (e.g., c-di-GMP) [3].

Three-component signal transduction systems are less frequent and comprise the sensor kinase and two cognate response regulators containing domains that enable DNA-binding and enzymatic functions [3,4,5,6,7]. The interconnection of three-component systems members is achieved on transcriptional and post-translational levels [4,5,6,7]. Although DNA-binding response regulators of three-component systems could stimulate the transcription of genes encoding the response regulators with enzymatic effector domains, these pair of regulators could also manifest the antagonistic activities (e.g., RocA1/RocR and BvgA/BvgR) [4,5]. So far, the involvement of these signal transduction systems in virulence regulation was described in pathogens such as *Pseudomonas aeruginosa* (RocS1A1R) [5], *Bordetella pertussis* (BvgAS/R) [4] and *Shewanella putrefaciens* (LrbS-LrbA-LrbR) [6].

Furthermore, the interconnection of various signal transduction pathways is required for some cellular processes, enabling the modulation of cell behavior in the most appropriate manner [3]. In addition, variation in stoichiometry, domain organization and cellular localization of sensor kinases contributes to the complexity of the signaling network [2].

*Pseudomonas* spp. is a nutritionally and metabolically versatile bacteria well adapted to wide range of ecological niches [8]. The successful adaptation of *Pseudomonas* spp. to fluctuating environmental conditions is mediated by numerous regulatory networks [1,9]. The signal transduction systems of genus *Pseudomonas* were best characterized in the opportunistic pathogen *P. aeruginosa*. Previous studies have shown that in *P. aeruginosa* signal transduction systems play a significant role in the regulation of virulence, including the biofilm formation, different types of motility, iron acquisition, production of fimbriae adhesins and type III secretion system, and in antibiotic resistance to aminoglycosides, carbapenems and polymyxins. Integrated complex signal transduction networks (two-, three- and multi-component systems) provide the quick and fine-tuned response of *P. aeruginosa* to changes in conditions within the host or in the environment [1,3,5,9,10]. The signal transduction systems are of importance for other representatives of genus *Pseudomonas* as well. In species *P. putida* TCS ColRS regulates not only the tolerance to phenol and different metals (zinc, iron, manganese and cadmium) [11,12], but also the Tn*4652* transposition during starvation [13]. Further, the regulatory role of TCS CzcRS in heavy metal resistance [14], RoxSR in redox signaling, plant colonization and organic molecule metabolism [15] as well as positive regulation of quorum sensing (QS) by GacA-GacS system was also reported [16]. In our previous study, the three-component system in *P. putida* WCS358 (RclSAR), in which sensor kinase RclS stimulates the *intI1* transcription in stationary growth phase, was described for the first time [7]. Apparently, this regulation appears to be rather indirect, at least through the expression regulation of stationary-phase alternative sigma factor RpoS [7].

In the present study, we identified the regulon of RclSAR system in *Pseudomonas capeferrum* WCS358 (previously known as *P. putida* WCS358 [17]) using Δ*rclS* mutant. RNA sequencing revealed the growth phase dependance of RclSAR signal transduction system activity as well as the regulation of diverse cellular activities such as RNA binding and metabolic and biocontrol processes by this signal transduction system. In addition, the virulence functions and resistance to antibiotics could be attributed to RclS regulon, directly or indirectly. Finally, we improved the interconnection model of the regulatory systems in *P. capeferrum* WCS358 previously proposed by Bertani and Venturi (2004) [16].

## 2. Results

### 2.1. RNA Sequencing and Mapping to the Reference Genome

In this study, the total RNA samples of the WCS358 wild type and Δ*rclS* mutant from the exponential (wt1 and mut1) and stationary growth phase (wt2 and mut2) were used for cDNA library preparation. Constructed cDNA libraries were subjected to Illumina sequencing and the obtained results are summarized in Table 1. The raw reads ranged between samples from 7,978,294 to 11,921,075, while after quality filtering clean data varied from 7,940,628 to 11,895,759 of reads. Since the base accuracy parameters Q20 and Q30 were over 98% and 95% for each library, respectively, the quality of the sequencing data was sufficient for further analysis. Additionally, the GC content ranged from 55.73% to 60.96%. The aligning of clean reads against genome of *P. capeferrum* WCS358 revealed that at least 97.84% reads were mapped. Uniquely mapped reads accounted for 96.27% to 98.41% of the total mapped reads (Table 1).

### 2.2. Gene Co-Expression and Differentially Expressed Genes (DEGs) Analysis

**Figure 1:**
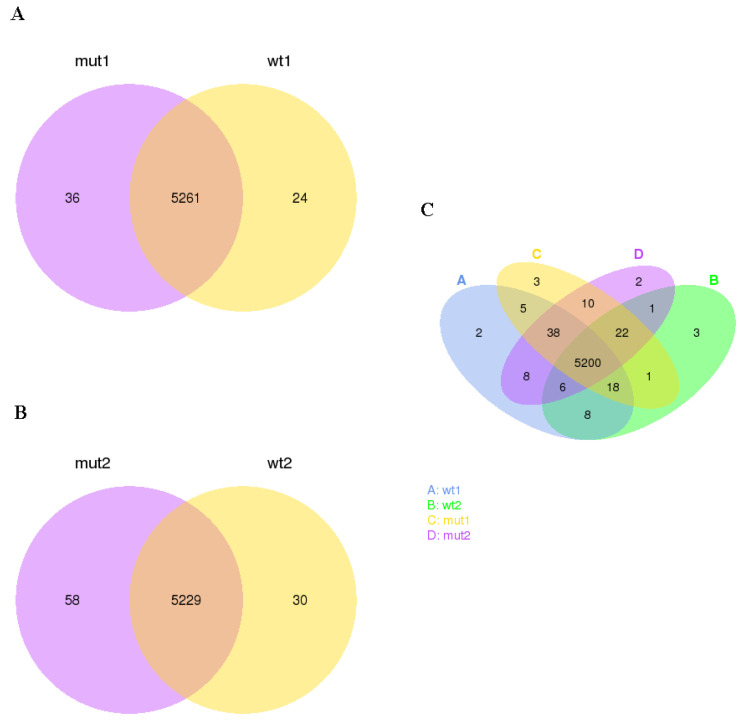
Gene co-expression. Venn diagrams depicted number of common and uniquely expressed genes in WCS358 wild type and Δ*rclS* mutant (**A**) in exponential growth phase; (**B**) in stationary growth phase; (**C**) in exponential and stationary growth phase.

The gene expression level of the tested samples was estimated by counting the reads that were mapped to the genes of the reference genome. Thereafter, the FPKM value of each gene was determined based on the length of the gene and sequencing depth and used as the final parameter of gene expression. Venn diagrams were constructed for visualization of the co-expressed and uniquely expressed genes in WCS358 wild type and Δ*rclS* mutant during the exponential and stationary phase of growth (Figure 1). We found the transcripts of 5261 and 5229 genes expressed in both strains throughout the exponential and stationary growth phase, respectively (Figure 1A,B). A number of uniquely expressed genes in WCS358 wild type was 24 and in Δ*rclS* mutant was 36 in the exponential phase of growth, while was 30 and 58 for wild type and Δ*rclS* mutant in the stationary growth phase, respectively. In addition, the co-expression of 5200 genes was determined in wild type and Δ*rclS* mutant strains in the exponential and stationary growth phase (Figure 1C).

**Figure 2:**
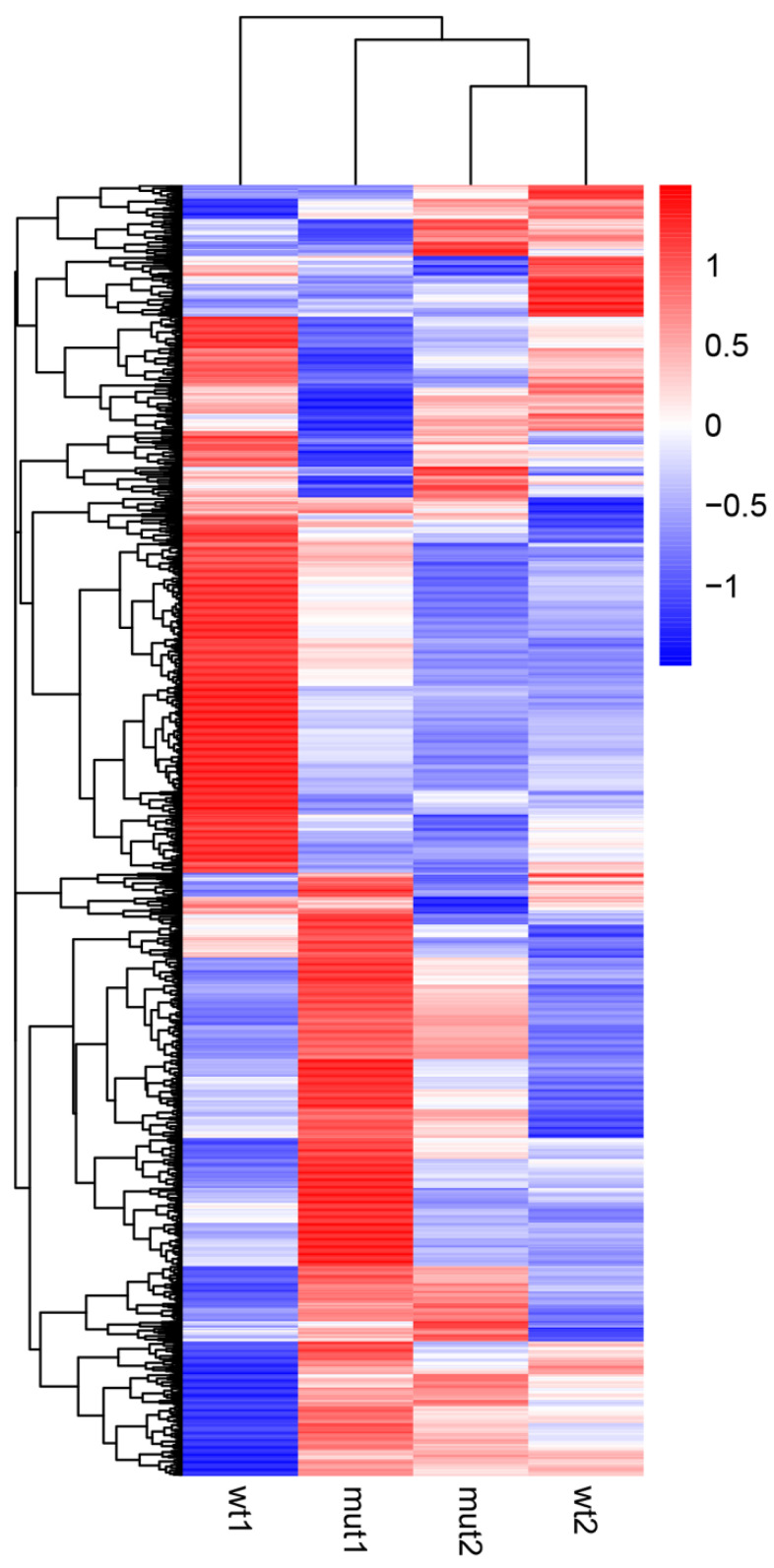
Hierarchical clustering of DEGs detected in WCS358 wild type and Δ*rclS* mutant in exponential and stationary growth phase. Scaled log2 expression values are presented in red and blue indicating high and low expressions, respectively.

Further, we examined differentially expressed genes (DEGs) comparing the WCS358 wild type and Δ*rclS* mutant using a fold change > 2 and corrected *p* value of 0.005 as the parameters of significance. A total of 1591 genes were identified as DEGs in the exponential growth phase, while the number of DEGs in the stationary growth phase was 574 (Figure S1). The number of up-regulated genes in Δ*rclS* mutant in the exponential (822) and stationary (384) growth phase was higher than the down-regulated genes in the exponential (769) and stationary (190) growth phase (Figure S1). Hierarchical clustering of DEGs in the wild type and Δ*rclS* mutant at the chosen two time points of growth revealed differential DEG expression profiles of wild type and Δ*rclS* mutant, with more prominent variation observed in the exponential growth phase (Figure 2). The lists of significant DEGs in Δ*rclS* mutant compared to wild type in the exponential and stationary growth phase are presented in Tables S1 and S2, respectively.

### 2.3. GO and KEGG Enrichment Analysis of DEGs

**Figure 3:**
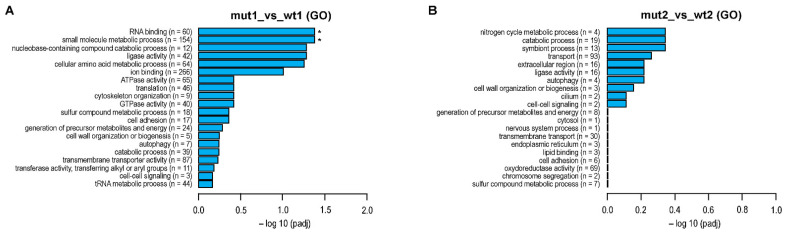
Summarized GO enrichment analysis of detected DEGs. The 20 most enriched GO terms are represented for exponential (**A**) and stationary (**B**) growth phase. The length of bar charts indicates statistical significance of each GO term (* *p* < 0.05).

The Gene Ontology (GO) enrichment analysis was performed to attribute the biological functions of detected DEGs. This analysis enabled categorization of DEGs into three GO groups: biological processes (BP), cellular components (CC) and molecular functions (MF). Figure 3 summarizes results obtained for all three GO categories (top 20 enriched GO terms). The highest number of DEGs in exponential growth phase was found to be involved in ion binding (n = 266), small molecule metabolic process (n = 154) and transmembrane transporter activity (n = 87), but after correction of *p* value only the RNA binding (MF) and small molecule metabolic process (BP) were recognized as statistically significant (Figure 3A). During the stationary phase of growth, neither of the detected functions were significant, but the transmembrane transport counted by far the largest number of DEGs (n = 93) (Figure 3B).

**Figure 4:**
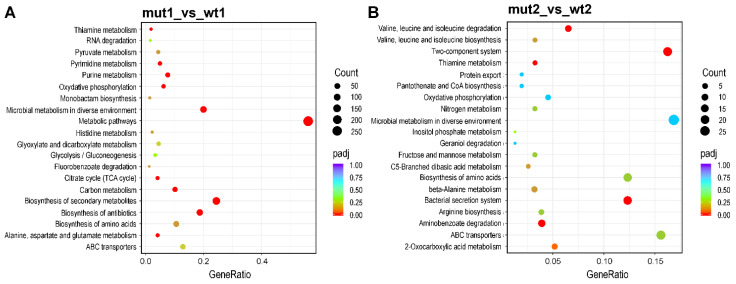
KEGG enrichment analysis of detected DEGs. The top 20 enriched KEGG pathways of DEGs in exponential (**A**) and stationary (**B**) growth phase. The x-axis represents the gene ratio, ratio of DEGs in pathway to the total number of annotated genes in the pathway. The value of gene ratio is proportional to the degree of enrichment. The size of the dots indicates the number of DEGs in each pathway, while the dot color represents corrected *p* values. The color vicinity to red is proportional to the enrichment significance.

The identification of pathways regulated by sensor kinase RclS was performed using KEGG (Kyoto Encyclopedia of Genes and Genomes) enrichment analysis. The 20 most significantly enriched pathways in the exponential and stationary phase of growth are shown in Figure 4. In the exponential growth phase 10 KEGG pathways (metabolic pathways, oxidative phosphorylation, microbial metabolism in diverse environments, biosynthesis of antibiotics, biosynthesis of secondary metabolites, purine metabolism, citrate cycle-TCA cycle, pyrimidine metabolism, alanine, aspartate and glutamate metabolism, carbon metabolism) were recognized as statistically significant (Figure 4A), while in the stationary growth phase only bacterial secretion system and aminobenzoate degradation showed corrected *p* value less than 0.05 (Figure 4B).

### 2.4. Validation of RNA Sequencing Using RT-qPCR

**Figure 5:**
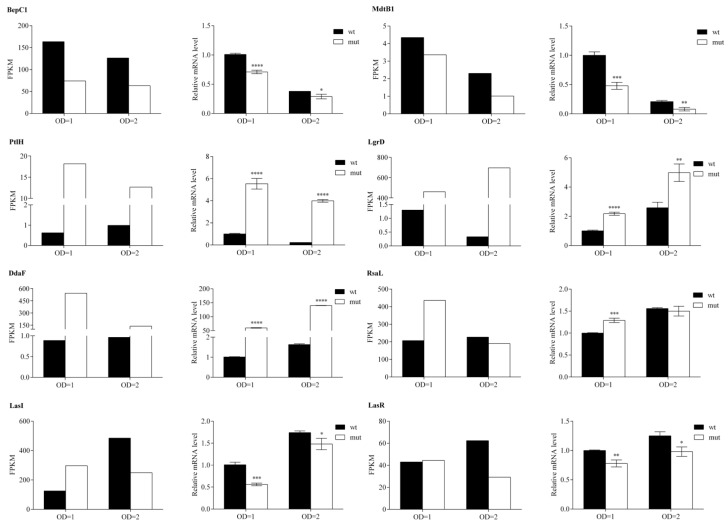
RT-qPCR based validation of RNA-seq analysis. Transcriptional level of eight selected genes in WCS358 wild type and Δ*rclS* mutant at two time points (OD = 1 and OD = 2) is represented as FPKM value (RNA-seq) and relative expression value normalized against those obtained for wild type in exponential growth phase (RT-qPCR). RT-qPCR data is represented as mean values ± standard deviations for three biological replicates. Student’s *t*-test was employed to compare differences between wild type and Δ*rclS* mutant measured by RT-qPCR (* *p* < 0.05; ** *p* < 0.01; *** *p* < 0.001; **** *p* < 0.0001).

In order to confirm RNA-seq reliability, the mRNA levels transcribed from eight selected genes (*bepC1*, *mdtB1*, *ptlH*, *lgrD*, *ddaF*, *rsaL*, *lasI* and *lasR*) were determined by RT-qPCR. The expression trend (up- and down-regulation) of the selected genes was comparable to those obtained from the RNA sequencing, indicating that our transcriptome analysis was valid (Figure 5). The only significant inconsistency was observed for the *lasI* gene mRNA levels during the exponential growth phase. The observed variations in gene expression intensity between RNA-seq and RT-qPCR could be rooted within the different sensitivity of the two methods applied here.

### 2.5. Antimicrobial Susceptibility of WCS358 Wild Type and ΔrclS Mutant

The differences in the susceptibility of WCS358 wild type and Δ*rclS* mutant to clinically relevant antimicrobials were determined, thus indicating the potential role of sensor kinase RclS in regulation of resistance to members of the different antibiotic classes (ceftazidime, gentamicin, novobiocin, levofloxacine and tetracycline) (Table 2). The results revealed that Δ*rclS* mutant had increased susceptibility to gentamicin, novobiocin, levofloxacine and tetracycline, and decreased susceptibility to ceftazidime compared to the wild type. MIC values for the ampicillin, chloramphenicol and erythromycin did not differ when wild type and Δ*rclS* mutant were compared.

### 2.6. AHLs, Pseudobactin 358 and Biofilm Production of WCS358 Wild Type and ΔrclS Mutant

**Figure 6:**
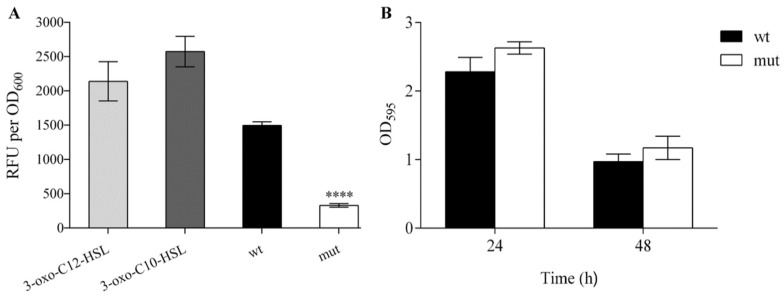
The effect of RclS knock-out to AHL production and biofilm formation in WCS358. (**A**) Production of AHLs by WCS358 wild type and Δ*rclS* mutant is represented as relative fluorescence units corrected for autofluorescence per unit of OD_600_ (RFU per OD_600_). Cell suspensions with 3-oxo-C10-HSL and 3-oxo-C12-HSL were used as a positive control. (**B**) Biofilm formation of WCS358 wild type and Δ*rclS* mutant was quantified by measuring absorbance at 595 nm after 24 and 48 h of incubation. The tests were performed in sextuplicate with three independent repeats. The results are represented as mean values ± standard deviations. Student’s *t*-test was employed to compare differences between wild type and Δ*rclS* mutant (**** *p* < 0.0001).

In order to compare the production of the quorum sensing (QS) signaling molecules (*N*-acyl-homoserine lactones, AHLs) between WCS358 wild type and Δ*rclS* mutant, fluorescence detection assay was performed. Reporter strain *P*. *putida* F117 (pKR-C12) was used for the detection of 3-oxo-C12-HSL and 3-oxo-C10-HSL. The obtained results indicted a significant decrease of AHLs production in Δ*rclS* mutant (approximately 4.5 times, **** *p* < 0.0001) relative to the wild type (Figure 6A). In contrast, the calculation of molar concentration of pseudobactin 358 from the wild type and Δ*rclS* revealed that the production of this siderophore was elevated in Δ*rclS* mutant by 17.7% (*** *p* < 0.001). Further, the statistically significant difference in biofilm formation was not observed between the wild type and Δ*rclS* mutant after 24 and 48 h (Figure 6B).

### 2.7. Cytotoxic Effect and Adhesion Ability of WCS358 Wild Type and ΔrclS Mutant

**Figure 7:**
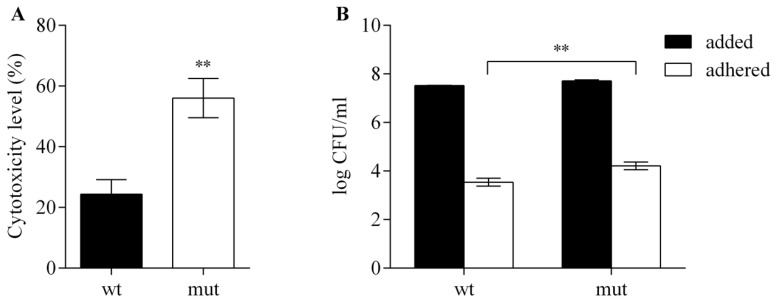
Cytotoxic effect and adhesion ability of WCS358 wild type and Δ*rclS* mutant using HaCaT keratynocite cell line as a model system. (**A**) Cytotoxicity level (%) of wild type and Δ*rclS* mutant measured by LDH assay was expressed relative to negative control (0%). (**B**) Adhesion ability of wild type and Δ*rclS* mutant was expressed as a ratio of adhered bacteria relative to added bacteria (log CFU/mL). The results are represented as mean values ± standard deviations for three biological replicates. Student’s *t*-test was employed to compare differences between wild type and Δ*rclS* mutant (** *p* < 0.01).

In order to reveal the pathogenic potential of WCS358 and role of the RclS sensor kinase in that phenotype, the cytotoxic effect and the ability of adhesion to the host cells were investigated in WCS358 wild type and Δ*rclS* mutant using the HaCaT keratinocyte cell line. Lactate dehydrogenase (LDH) assay revealed significant increase in the cytotoxic potential of Δ*rclS* mutant comparing to the wild type (approximately 2.3 times), although WCS358 wild type also had significant cytotoxic effect relative to negative control (Figure 7A). The adhesion assay showed that both WCS358 wild type and Δ*rclS* mutant have low adhesion ability to HaCaT keratinocyte cell line (<0.1%). However, it was notable that Δ*rclS* mutant have significantly increased adhesion capability relative to wild type (3.57 times) (Figure 7B).

## 3. Discussion

The rapid and appropriate physiological response to fluctuating environmental conditions has been recognized as a key factor of bacterial cell adaptation and survival [1]. Signal transduction systems are the major players in this process enabling sensing of diverse environmental stimuli and response through regulating cellular changes [2]. Virulence and antibiotic resistance of clinically important pathogens are also regulated through the signal transduction systems resulting in the recognition of these systems as potential novel antimicrobial targets [1,9,18].

**Figure 8:**
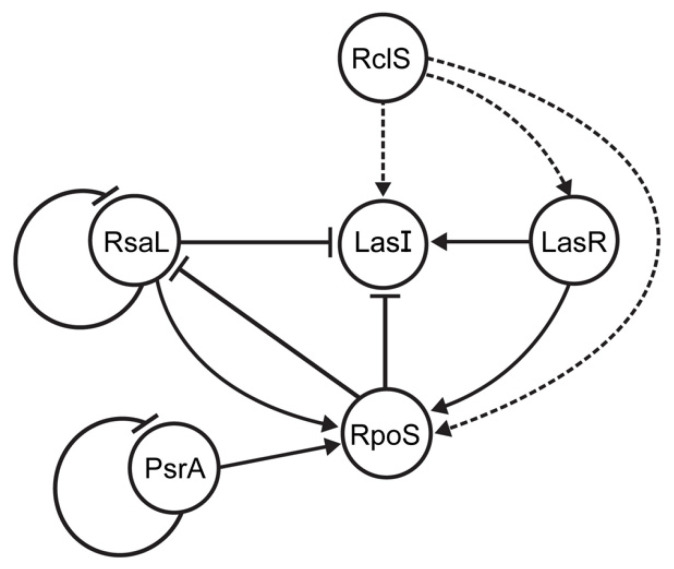
Improved interconnection model of *P. capeferrum* WCS358 regulatory systems (RclSAR, LasIR and RpoS) in stationary growth phase (improvement of model proposed by Bertani and Venturi (2004) [16], adapted with permission). Triggering of signal transduction pathway starting with RclS activation leads to increased expression of the *lasI*, *lasR* (this study) and *rpoS* genes [7]. Gene regulation network between RpoS, LasIR (PpuIR), RsaL and PsrA proteins were described in previous study [16]. Dashed lines indicate indirect gene regulation by RclS sensor kinase.

The outstanding metabolic versatility and adaptability of pseudomonads to changing habitats pinpointed these bacteria as attractive models for signal transduction system research [1,9]. Although signal transduction systems of *P. aeruginosa* are the best characterized due to their clinical relevance, *P. putida* was also subject of this type of study. Since *P. putida* is typically soil and/or aquatic bacterium, the focus of the research was on TCSs enabling tolerance to pollutants and plant colonization [11,12,14,15,19]. Albeit rarely, *P. putida* could also cause different types of infections in human beings [20,21,22]. Of special importance was often described multidrug-resistant phenotype of these isolates [20,21,22], while the pathogenic potential was isolate-specific [23]. Additionally, *P. putida* has been recognized as a potential reservoir of antibiotic resistance determinants that could be transferred to more successful clinical pathogens [24]. As aforementioned, our previous study investigated the regulation of the *intI1* gene transcription, since integrase class 1 performs a key role in dissemination of antibiotic resistance cassettes located on integrons [7]. We observed that RclS sensor kinase, a member of the RclSAR three-component system, has been characterized as the activator of the *intI1* transcription in the stationary phase of growth in *P. putida* WCS358 (now *P. capeferrum* WCS358) [7]. Since this was the first described three-component system in *P. capeferrum*, the RNA sequencing was employed to determine the potential regulon of RclSAR system, and to examine the growth phase dependence of regulon expression. This work showed the significant change in the number of uniquely expressed genes in Δ*rclS* mutant compared to the wild type underlying the importance of RclS signal pathway not only for fine tuning of expression, but also for the ON/OFF general expression regulation. Further, the results obtained from DEG analysis indicate that the regulatory potential of the signal transduction system starting with RclS sensor kinase on gene expression is more pronounced in the early rather than the late stages of growth, with a more prominent activation of gene transcription in Δ*rclS* mutant strain. The results of this study indicate that the regulation mediated by RclS was growth phase dependent. The growth phase dependent variation in activity and expression of TCSs was previously described as well [25,26]. Although speculative, the more pronounced up-regulation of gene transcription in Δ*rclS* strain could be explained through the elevated level of DNA-binding response regulator RclA in mutant strain and consequential activation of target genes [7]. GO enrichment analysis, a method for defining biological functions of detected DEGs, revealed that RclS sensor kinase is included in the regulation of DEGs classified in RNA binding GO term. This observation is of special importance because it leads to the conclusion that RclS sensor kinase, besides regulation on transcriptional level, could potentially act as a post-transcriptional regulator of gene expression. Moreover, the detection of pathways regulated by RclS indicates its contribution to the diverse metabolic and biocontrol processes regulation in the exponential phase of growth. As noted in this study, the regulation of amino acid, nucleotide and carbon metabolism mediated by TCS was described in previous studies. This phenomenon has been observed in important pathogens such as *Bacillus anthracis* (WalRK) [27] and uropathogenic (KguS/KguR) [28] and enterohemorrhagic *Escherichia coli* (QseBC) [29]. Moreover, Sonawane and colleagues (2006) [30] reported the involvement of AauR-AauS TCS in uptake and metabolism of acidic amino acids in *P. putida* KT2440. The assumed RclS role in antibiotic production regulation is not a single example of signal transduction system involvement in the modulation of antibiotic biosynthesis. In various antibiotic producers, predominantly members of the genus *Streptomyces*, diverse TCSs control the biosynthesis of secondary metabolites, including antibiotics [31]. For example, in actinomycetes *Nonomuraea gerenzanensis* and *Saccharopolyspora erythraea* (formerly known as *Streptomyces erythraeus*) TCS negatively regulates the production of glycopeptide A40926 and erythromycin, respectively [32,33]. Besides mapping the RclS regulon genes by transcriptome analysis, our aim was the phenotypic determination of RclS sensor kinase influence on the pathogenic potential of *P. capeferrum* WCS358. From one side, the obtained results hinted that RclS leads to an elevated resistance to the members of the different antibiotic classes (aminocoumarins, aminoglycosides, fluorohinolones and tetracyclines), while resistance to β-lactam ceftazidime was suppressed. Similarly, as shown in this study, *P. aeruginosa* isolates containing mutations in the *pmrB* gene, the gene encoding for sensor kinase PmrB, were more susceptible to various antibiotics (e.g., ciprofloxacin and gentamicin) [34]. Contrary to that, inactivation of the *gluS* and *gluR* genes provided resistance to β-lactams in *Burkholderia glumae* [35]. On the other hand, we investigated the impact of RclS mediated signal transduction pathway to virulence of WCS358. Since AHL-dependent QS regulates bacterial virulence, and TCSs could regulate its expression [16], we monitored AHLs production in WCS358 wild type and Δ*rclS* mutant. The production of AHLs has been significantly decreased in Δ*rclS* mutant indicating positive regulation of QS system mediated by RclS sensor kinase. In agreement with this, RNA-seq and RT-qPCR confirmed that mRNA levels of AHLs synthase LasI and regulator LasR were reduced in the mutant strain during the stationary phase of growth independently of its repressor RsaL mRNA levels (Figure 5). Similar to the noted interconnection of the three global regulatory systems (GacA-GacS TCS, RpoS and PpuIR QS) described by Bertani and Venturi (2004) [16], we observed a close link among RclS sensor kinase, RpoS and LasIR (PpuIR) QS system [7]. Accordingly, we proposed the improved interconnection model of regulatory systems in *P. capeferrum* WCS358 designated by Bertani and Venturi (2004) [16] (Figure 8). In contrast to the commonly described role of QS in biofilm formation, in WCS358 the absence of AHL production caused by the *lasI* (*ppuI*) gene mutagenesis did not affect biofilm formation [36]. Our result that showed no difference between WCS358 wild type and Δ*rclS* mutant in biofilm formation supports this observation. The statistically significant increase of pseudobactin 358 production in Δ*rclS* mutant indicates the negative regulation of its production mediated by sensor kinase RclS. Previously, the role of FleS/FleR TCS in siderophore biosynthesis regulation in *P. aeruginosa* was described [37]. Moreover, this TCS regulates the secretion systems in *P. aeruginosa* [37], and a similar result has been obtained by KEGG analysis for RclSAR system of WCS358 in the stationary phase of growth. This observation, as well as the results obtained from the cytotoxicity and adhesion tests performed on the keratinocyte cell line, indicates the role of RclS in *P. capeferrum* WCS358 virulence potential. Although *P. capeferrum* WCS358 was known as a plant-beneficial strain, it is also recognized as a causative agent of skin infection [17]. Thus, RclS sensor kinase could act as a silencer of pathogenic behavior. Similar to our results, Bricio-Moreno and colleagues (2018) [34] reported that *P. aeruginosa* isolates with mutation in the *pmrB* gene of TCS showed increased adherence to airway epithelial cells. Additionally, the *pmrB* gene mutation enhanced the persistence of the described isolates in the murine lungs [38]. Furthermore, the potential role of the response regulator RclR with phosphodiesterase activity in WCS358 virulence should not be neglected [7]. Thus, RclR could affect WCS358 virulence indirectly, through the degradation of c-di-GMP, a second messenger involved in the regulation of bacterial virulence. A recent study summarizing the contribution of c-di-GMP in bacterial virulence revealed that the role of this second messenger is specific for each pathogen, strain as well as type of infection [39]. Above all, the possibility that RclSAR three-component system could cross-talk with other signal transduction pathways in *P. capeferrum* WCS358 and consequently be involved in the complex regulation of the cellular processes remains open, as previously was noticed for other signal transduction systems [40].

## 4. Materials and Methods

### 4.1. Bacterial Strains and Chemicals

Strains *P. capeferrum* WCS358 (formerly *P. putida* WCS358) wild type and *P. capeferrum* WCS358 Δ*rclS* mutant with inactivated gene encoding RclS sensor kinase [7] were used in this study. Biosensor strain *P*. *putida* F117 was used for AHL detection [41]. AHLs *N*-3-oxodecanoyl-L-homoserine lactone (3-oxo-C10-HSL) and *N*-3-oxododecanoyl-L-homoserine lactone (3-oxo-C12-HSL) were purchased from Sigma-Aldrich. AHL stock solutions (0.5 mM) were prepared in dimethyl sulfoxide (DMSO) and stored at −20 °C for biological assays.

### 4.2. RNA Extraction and Sequencing

*P. capeferrum* WCS358 wild type and Δ*rclS* mutant were incubated in cation-adjusted Mueller Hinton (caMH) medium at 30 °C with aeration until reaching OD_600_ value of 1 and 2 (Plate Reader Infinite 200 pro, MTX Lab Systems, Midland, ON, Canada). RNA extraction from collected bacterial cells was performed using RNeasy Mini Kit (Qiagen, Hilden, Germany), with a modified lysis step [42]. AmbionDNAfree Kit (Thermo Fisher Scientific, Waltham, MA, USA) was used for DNase I treatment. The total RNA concentration and purity was determined with NanoPhotometer spectrophotometer (IMPLEN, München, Germany), while RNA integrity was confirmed using Agilent 2100 Bioanalyzer (Agilent Technologies, Santa Clara, CA, USA). After cDNA library construction, the sequencing was performed using Illumina platform. The cDNA library preparation and RNA-seq was carried out by Novogene Bioinformatics Technology Co., Ltd., Beijing, China.

### 4.3. Data Analysis

The raw reads were filtered to remove low quality reads and reads containing adapters and poly-N sequences. Further, the clean reads were used for calculating Q20, Q30 and GC content. Moreover, clean reads were aligned to genome of *P. capeferrum* WCS358 strain that was used for RNA isolation in this work (JAKNRD000000000) by Bowtie2 [43]. HTseq was applied to count the number of reads mapped to each gene and FPKM (Fragments PerKilobase of transcript sequence per Millions base pairs sequenced) value of each gene was determined based on the length of the gene and sequencing depth. For co-expression analysis, the threshold of the FPKM value was set to 0.3. Differential expression analysis of wild type and mutant strain was assessed using DEGSeq R package, while *p* values were adjusted according to Benjamini & Hochberg method. The genes expressed with a fold change > 2 and corrected *p* value of 0.005 were considered as significant differentially expressed genes (DEGs). Heat map was employed to cluster DEGs detected in wild type and Δ*rclS* mutant in exponential and stationary phase of growth.

### 4.4. GO and KEGG Enrichment Analysis

The GO (http://www.geneontology.org/, accessed on 1 April 2019) enrichment analysis of DEGs was applied using GOseq R package, in which the correction of gene length bias was performed. The alternations in certain metabolic or signal transduction pathways in the mutant strain relative to the wild type were determined using KEGG (http://www.genome.jp/kegg/, accessed on 1 April 2019) enrichment analysis. KOBAS software was used for testing the statistical enrichment of DEGs in KEGG pathways. GO and KEGG terms with corrected *p* < 0.05 were considered significantly enriched by DEGs.

### 4.5. Real Time Quantitative PCR (RT-qPCR) Analysis

The validating of the transcriptome sequencing analysis was performed by means of RT-qPCR for eight selected genes. After RNA isolation and DNase I treatment, reverse transcription was done with a RevertAidRT Reverse Transcription Kit (Thermo Fisher Scientific) according to the manufacturer’sprotocol. Primers used for RT-qPCR are listed in Table S3. RT-qPCR was carried out with a FastGene 2x IC Green Universal ROX (Nippon Genetics Europe GmbH, Düren, Germany) in a 7500 RealTime PCR System thermocycler (Applied Biosystems, Thermo Fischer Scientific). Thermal conditions comprised an initial denaturation at 95 °C for 2 min, 40 cycles at 95 °C for 5 s and 60 °C for 32 s. Normalization was performed against the *rpoD* gene using the 2^−ΔΔ*CT*^ method (relative) [44]. The values obtained for Δ*rclS* mutant were then normalized against those acquired for WCS358 wild type. RT-qPCR experiments were done in triplicate.

### 4.6. Antimicrobial Susceptibility Testing

The antibiotic susceptibility was determined for WCS358 wild type and Δ*rclS* mutant by microdilution method according to the European Committee on Antimicrobial Susceptibility Testing (http://www.eucast.org, accessed on 1 April 2019). The strains were tested against ampicillin (100–800 µg/mL), ceftazidime (2.5–20 µg/mL), chloramphenicol (20–160 µg/mL), erythromycin (100–800 µg/mL), gentamicin (2.5–20 µg/mL), novobiocin (100–800 µg/mL), levofloxacine (0.625–5 µg/mL) and tetracycline (2.5–20 µg/mL).

### 4.7. Preparation of Crude Ethyl Acetate Extract

*P*. *capeferrum* WCS358 wild type and Δ*rclS* mutant bacterial cultures (one liter) were cultivated in M9 minimal medium (10×; 33.7 mM Na_2_HPO_4_ × 7H_2_0, 22 mM KH_2_PO_4_, 8.55 mM NaCl, 9.35 mM NH_4_Cl, 1 mM MgSO_4_ and 0.3 mM CaCl_2_) supplemented with 0.5% glucose for 24  h at 30 °C, aerobically. After centrifugation at 13,680*× g* at 4 °C for 30  min, the collected supernatants were mixed with an equal volume of ethyl acetate and 0.1 mL/l glacial acetic acid (Sigma-Aldrich, St. Louis, MI, USA). The AHL extraction from supernatant was performed twice with a vigorous shaking (30  min at room temperature). Extracts were dried by evaporation using a vacuum rotary evaporator at 30 °C (Buchi Rotavapor, R200, Fisher Scientific) and the dry mass was dissolved in DMSO for further analysis [45].

### 4.8. Fluorescence Assay

The overnight culture of the reporter strain *P*. *putida* F117 (pKR-C12) was diluted to the optical turbidity equivalent to 0.5 McFarland standard, after which it was ten times additionally diluted and incubated with WCS358 wild type and Δ*rclS* AHL extracts (1 mg/mL, final concentration). Cell suspensions with 3-oxo-C10-HSL and 3-oxo-C12-HSL (5 μM, final concentration) were used as a positive control. Fluorescence assay was monitored in black-walled clear-bottom 96-well microplates (Thermo Fisher Scientific). After 6 h of incubation (30 °C, 70 rpm), the green fluorescence (an excitation/emission wavelength detection at 474/515 nm) and cell density (OD_600_) were simultaneously measured with a Plate Reader Infinite 200 pro (MTX Lab Systems). The background fluorescence of the AHL biosensor strain was determined by incubation of the biosensor with caMH medium as a negative control. Specific fluorescence is defined as the relative fluorescence units corrected for autofluorescence per unit of OD_600_ [41]. The test was performed in sextuplicate with three independent repeats.

### 4.9. Biofilm Formation Assay

The biofilm formation ability of WCS358 wild type and Δ*rclS* mutant was performed according to the method described by Stepanović et al. (2007) [46], with modifications. Briefly, wells of 96-well microtiter plates (Sarstedt) were filled with 180 μL caMH broth medium and 20 μL of WCS358 wild type and Δ*rclS* overnight cultures (adjusted to the 0.5 McFarland standard) were added. Bacterial cultures were incubated aerobically for 24 or 48 h at 30 °C. Sterile medium was used as a negative control. After incubation and washing (three times with 1X phosphate-buffered saline, PBS), bacteria were fixed by drying at 65 °C for 30 min. Staining and visualization of biofilm was performed using 0.1% crystal violet (HiMedia Labs Pvt. Ltd., Mumbai, India) (30 min at room temperature). The stain was rinsed by washing three times with 1X PBS and then resolubilized with 96% ethanol and acetone (4:1). Quantification of biofilm formation was determined by measuring absorbance at 595 nm using Plate Reader Infinite 200 pro (MTX Lab Systems). Both strains were tested in sextuplicate with three independent repeats.

### 4.10. Isolation of Siderophore Pseudobactin 358

WCS358 wild type and Δ*rclS* mutant were cultivated in succinate medium [47] for 48 h at 30 °C, aerobically. After cultivation, the OD_600_ values of cultures were measured and bacterial cells were pelleted by centrifugation (4500*× g*, 2 min). Further, optical density at 400 nm was determined for obtained supernatants (Plate Reader Infinite 200 pro, MTX Lab Systems). Molar concentration of isolated pseudobactin 358 was calculated using the following equation:

C = A/ε × L

where A represented OD_400_ value of supernatant, ε extinction coefficient specific for pseudobactin 358 and L optical path length. The experiment was done in triplicate.

### 4.11. Cytotoxicity and Adhesion Assays

The cytotoxic capacity of WCS358 wild type and Δ*rclS* mutant was determined using human keratinocyte HaCaT cell line model system. HaCaT keratinocyte was cultivated in high glucose DMEM, supplemented with 2 mM L-glutamine, 10% fetal bovine serum, 100 U/mL penicillin and 100 μg/mL streptomycin (Gibco Life Technologies, Waltham, MA, USA). Prior to treatment, HaCaT cells (2 × 10^5^ cells/well) were plated in 24-well plates (Sarstedt) and incubated overnight at 37 °C with 5% CO_2_. Before adding the bacterial cells, the cells were washed in 1X PBS and suspended in the aforementioned medium without antibiotics.

WCS358 wild type and Δ*rclS* mutant were cultivated in caMH broth at 30 °C with shaking overnight. The cells (2 × 10^7^ CFU/mL) were harvested and washed in 1X PBS. After centrifugation (4500*× g*, 5 min), the obtained pellets were suspended in high glucose DMEM containing 2 mM L-glutamine and 10% FBS.

HaCaT keratinocyte cells were infected with WCS358 wild type or Δ*rclS* by addition of the bacterial cell suspension to a 24 well plate containing HaCaT cells (multiplicity of infection was 100). For cytotoxicity assay, co-incubation was performed at 30 °C with 5% CO_2_ for 24 h. Cytotoxicity level of analyzed strains was monitored by LDH Cytotoxicity Assay Kit according to the manufacturer’s protocol (Thermo Scientific). Non-treated HaCaT cells represented negative control. For adhesion assay, HaCaT cells were infected with bacterial cells at 30 °C with 5% CO_2_ for 1 h. After washing (three times with 1X PBS) to remove unattached bacteria, cells were treated with trypsin/EDTA solution (Gibco Life Technologies) to collect adherent bacterial cells. Serial dilutions of bacteria before and after adhesion assay were prepared and used for determination of adherent cells number by plating on Luria Bertani (LB) agar plates (30 °C, 24 h). The adhesion potential of WCS358 wild type and Δ*rclS* was expressed as ratio of adhered bacteria relative to number of added bacteria. The experiment was done in triplicate.

### 4.12. Statistical Analysis

The obtained results are presented as mean values ± standard deviations. Student’s *t*-test was performed to compare differences between WCS358 wild type and Δ*rclS* mutant strain. Values at *p* 0.05 or less were considered to be statistically significant.

## 5. Conclusions

This study enabled the identification of the three-component system RclSAR regulon in *Pseudomonas capeferrum* WCS358 using Δ*rclS* mutant. Transcriptome analysis revealed that the expression regulation mediated by sensor kinase RclS is growth phase dependent and more pronounced in the early rather than the late stages of growth. Enrichment analyses showed that RclS has been included in the regulation of the diverse metabolic and biocontrol processes in *P. capeferrum* WCS358. Moreover, phenotypic tests revealed that RclS sensor kinase mostly contributes to the elevation of antibiotic resistance (gentamicin, novobiocin, levofloxacine and tetracycline) as well as the production of AHLs, enhancing survival and intercommunication of WCS358 cells. In addition, the inactivation of the gene encoding RclS sensor kinase leads to increased levels of virulence factor production (such are siderophores and adhesion to host cells). Finally, this study enabled a more comprehensive understanding and the creation of a model of regulatory networks interconnection in *P. capeferrum* WCS358.

## Figures and Tables

**Table 1:** Summary of RNA-seq data.

Samples	wt1	mut1	wt2	mut2
Raw reads	7,982,150	11,145,045	7,978,294	11,921,075
Clean reads	7,940,628	11,117,800	7,965,543	11,895,759
Q20 (%)	98.42	98.37	98.42	98.46
Q30 (%)	95.24	95.11	95.14	95.22
GC content (%)	60.96	57.76	55.87	55.73
Total mapped (%)	97.84	98.79	98.87	99.02
Uniquely mapped (%)	96.27	98.00	98.17	98.41

**Table 2:** Antimicrobial susceptibility profiles of WCS358 wild type and Δ*rclS* mutant.

Antibiotic	MIC (μg/mL)	
	Wt	Mut
Ampicillin	>800	>800
Ceftazidime	5	10
Chloramphenicol	80	80
Erythromycin	800	800
Gentamicin	5	2.5
Novobiocin	800	400
Levofloxacine	1.25	0.625
Tetracycline	10	5

## Supplementary Materials

The supporting information can be downloaded at: https://www.mdpi.com/article/10.3390/ijms23158232/s1. Reference [7] is cited in the supplementary materials.
