# Supplementary material for: RclS Sensor Kinase Modulates Virulence of Pseudomonas capeferrum
Source: Int J Mol Sci. 2022 Jul 26;23(15):8232. doi: 10.3390/ijms23158232 (PMC9331949; doi:10.3390/ijms23158232)
Supplement: Supplementary file 1 [file ijms-23-08232-s001.zip › Table S3.pdf]

Table S3. Primers used in RT-qPCR

Primer	Sequence (5'-3')	Length (bp)	Reference
BepC1 Fw	GCGTGGCGGCTACTACCCTTCCG	169	This study
BepC1 Rev	GCCGTCGAACAGCATCTGGCGC		
MdtB1 Fw	CGGAAACCATGGCCTCGTCGG	178	
MdtB1 Rev	GCGGTGTTGATCGCGGCCTGG		
PtlHFw	GGGTAAGCCAATGCAACACATCGG	187	
PtlH Rev	GGCGAACGTGGCGGCGCGGACC		
LgrDFw	CGGTGTACTGGGCGATACCC	140	
LgrD Rev	GGGACAGGCCCATGTGCG		
DdaFFw	GCGTTAAGCGGTTGTCTGAAGC	165	
DdaF Rev	GCGCATCGGAAAACTTAATACCC		
RsaLFw	CGCCTGGGCCTCAAGCAGAGCC	154	
RsaL Rev	GGTCGCTTTCCTCCAGGCGACCGC		
LasIFw	GCGCAACGCCACCGCACGCTCG	153	
LasI Rev	GCGATCGTCAGCTATGCC		
LasRFw	CCGCCATCTGGCGCGAGCTGACC	169	
LasR Rev	CCACACCCGTGCACCTGACGAGC		
RpoDFw	GCGTGAAATGGGTACTGTCGAGC	134	[7]
RpoD Rev	CGCTGAGAATGTAGTCG		
