# Supplementary figures and images for: RclS Sensor Kinase Modulates Virulence of Pseudomonas capeferrum
Source: Int J Mol Sci. 2022 Jul 26;23(15):8232. doi: 10.3390/ijms23158232 (PMC9331949; doi:10.3390/ijms23158232)

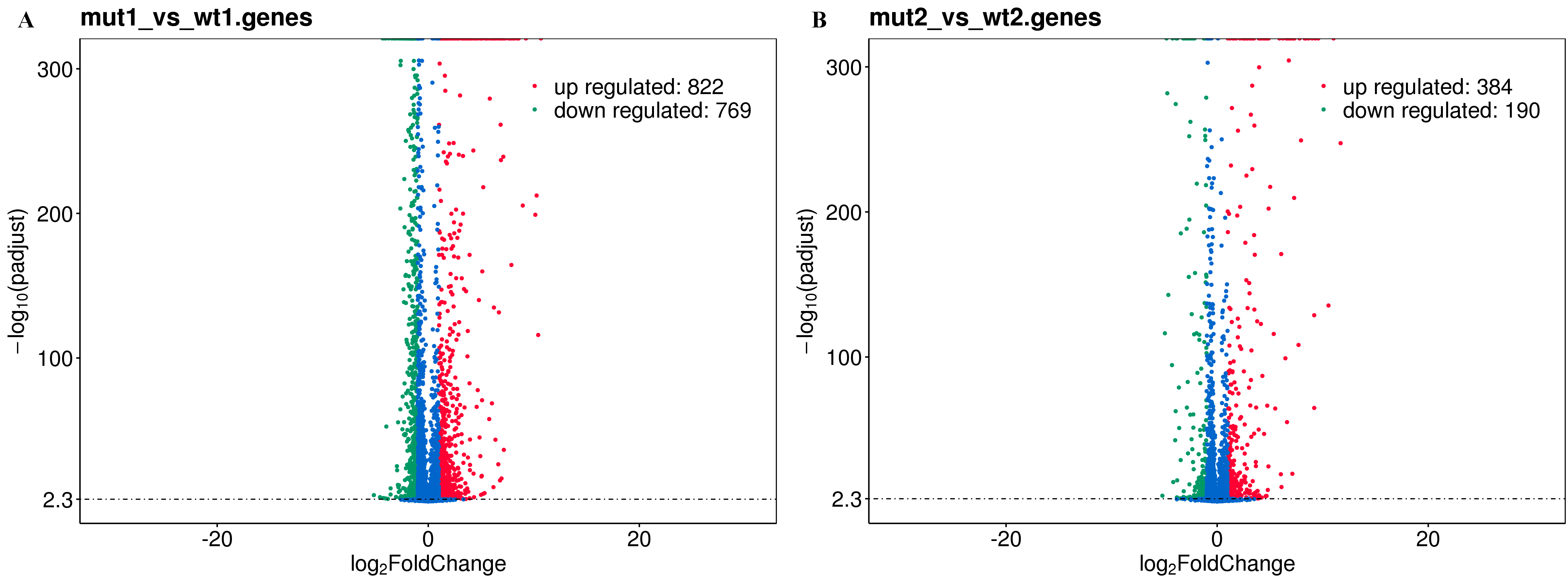

Supplement: Supplementary file 1 [file ijms-23-08232-s001.zip › Figure S1.TIF.tif]
